# Peritoneal Carcinomatosis of Malignant Gynecological Origin: A Systematic Review of Imaging Assessment

**DOI:** 10.3390/jcm13051254

**Published:** 2024-02-22

**Authors:** Rosaria Meucci, Daniela Prosperi, Chiara Lauri, Giuseppe Campagna, Pallavi Nayak, Francesco Garaci, Alberto Signore

**Affiliations:** 1 Nuclear Medicine Unit, Department of Medical-Surgical Sciences and of Translational Medicine, Faculty of Medicine and Psychology, “Sapienza” University, 00189 Rome, Italy; chiara.lauri@uniroma1.it (C.L.); gius.campagna@gmail.com (G.C.); pallavi.nayak@uniroma1.it (P.N.); alberto.signore@uniroma1.it (A.S.); 2 U.O.C. Diagnostic Imaging, PTV Policlinico “Tor Vergata” University, Viale Oxford 81, 00133 Rome, Italy; garaci@gmail.com; 3 Nuclear Medicine Unit, University Hospital Sant’Andrea, 00189 Rome, Italy; dprosperi@ospedalesantandrea.it; 4 Department of Medical and Surgical Sciences and Translational Medicine, Ph.D. School in Translational Medicine and Oncology, Faculty of Medicine and Psychology, Sapienza University of Rome, 00189 Rome, Italy

**Keywords:** ovarian cancer, endometrial cancer, PCI, peritoneal carcinomatosis, computerized tomography, MRI, FDG, PET/CT, PET/MRI

## Abstract

This systematic review, conducted using the Preferred Reporting Items for Systematic Reviews and Meta-Analyses (PRISMA) protocol, aims to comprehensively assess the current state of the art of imaging modalities for the evaluation of peritoneal carcinomatosis arising from malignant gynecological origins, with a focus on ovarian and endometrial cancers. A systematic search of relevant databases was performed, adhering to predetermined inclusion and exclusion criteria. Studies reporting the use of computed tomography (CT), magnetic resonance imaging (MRI), fluorodeoxyglucose (FDG) positron emission tomography (PET), PET/CT, and PET/MRI in the assessment of peritoneal carcinomatosis from gynecological malignancies were included. The review encompasses an overview of selected studies, highlighting the strengths and limitations of each imaging modality in diagnosing and characterizing peritoneal carcinomatosis. Overall, a wide variability in the reported accuracy of different imaging techniques emerges from literature, mainly due to the type of the study, technical issues, and patient characteristics. Although a meta-analysis could not be performed due to a scarcity of data, this systematic review provides valuable insights into the several imaging approaches used in peritoneal carcinomatosis of gynecological origin. The findings aim to inform clinical decision making and guide future research endeavors in this critical aspect of gynecological oncology.

## 1. Introduction

### 1.1. Peritoneal Carcinomatosis: An Insight

Peritoneal carcinomatosis (PC) is a deadly neoplastic condition with a frequently unfavorable prognosis. Characterized by metastatic infiltration of the peritoneum, a serous membrane enveloping the abdominal viscera, this condition poses significant challenges regarding therapeutic interventions and clinical outcomes [1].

Ovarian and gastric carcinomas are the most likely to spread to the peritoneum. Additionally, peritoneal deposits may arise from the pancreas, breast, appendix, biliary tract, liver, lung, and genitourinary tract malignancies [2].

**Figure 1:**
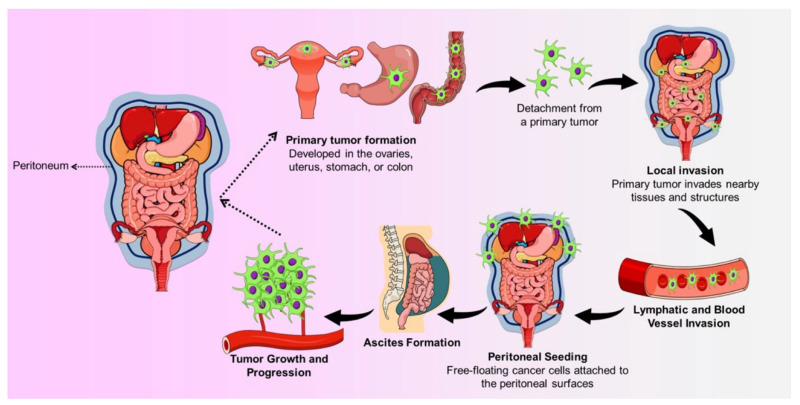
The development of peritoneal carcinomatosis.

The development of a PC involves several steps: It starts by detaching cancer cells from early tumors. These detached cells then stick to peritoneal mesothelial cells, making them shrink and reveal the underlying basement membrane. The cancer cells then multiply, forming a cluster. This cluster promotes the growth of new blood vessels (angiogenesis). The combination of the cluster and the surrounding blood vessels supports the tumor’s continued growth [3], as illustrated in Figure 1.

### 1.2. Peritoneal Cancer Index (PCI) and Radiological Impact on Therapeutic Planning

Cytoreductive surgery (CRS) aims at removing visible peritoneal implants by performing a peritonectomy and multiple organ resections. In the past few decades, laparoscopy has rapidly become a valid alternative to laparotomies, being a less invasive tool for both diagnostic and therapeutic purposes. Indeed, it allows the visualization of the peritoneal space, the assessment of metastases, and the possibility of performing biopsies, administering intraperitoneal chemotherapy, and assessing its efficacy during follow-up. Nevertheless, laparoscopic procedure may be also associated with some complications, although these are rarely life-threatening [2]. Radiologic and Nuclear Medicine imaging have great potential for assessing the entity of PC and for providing complementary information for surgeries.

**Figure 2:**
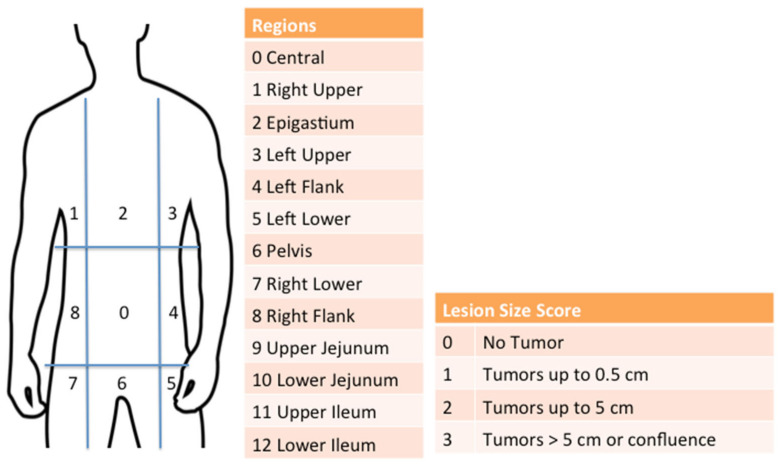
Peritoneal Cancer Index.

PC is a frequently encountered condition in radiologists’ daily practice. A comprehensive understanding of peritoneal anatomy, physiology, seeding mechanisms, potential differential diagnoses, and imaging findings associated with PC can significantly enhance the quality of radiology reports, thus having a significant impact on therapy decision making. The Peritoneal Cancer Index (PCI), developed by Paul Sugarbaker in 1996 [4], has rapidly become a crucial tool for outcome prediction and for deciding whether a patient is a suitable candidate for cytoreductive surgery (CRS) or neoadjuvant chemotherapy (NACT) before surgery and for estimating the potential benefits of the procedure [5,6]. Indeed, regardless of the origin and the histotype of the primary tumor, this scoring system has been recognized as the standard for PC assessment, being the most reliable and reproducible method capable of quantifying PC and of providing important prognostic information. According to this classification, the abdomen is divided into nine regions and the small bowel into four additional sectors. The total score is derived from the sum of the lesion size scores for each region (Figure 2) [7]. Coccolini et al. identified a PCI cut-off of 12 for obtaining the best survival benefit in patients with PC arising from gastric cancer [8]. Nevertheless, the optimal PCI cut-off for attempting a complete cytoreduction (CC), balancing the risks and benefits of each approach, has not been standardized yet [8,9].

The completeness of cancerous tissue removal is crucial for improving the life expectancy and quality of life of patients with PC; therefore, in recent years, several strategies and combinations of surgery and chemotherapeutic schemes have been developed and optimized to improve survival for gynecological cancers, as well as for tumors of other origins [10,11]. Many trials in recent years have demonstrated the benefits of NACT before surgery in colorectal and gastric cancer to allow tumor downstaging and complete CC during subsequent surgery [12,13].

Identifying preoperatively patients who are candidates for NACT, rather than laparotomy using non-invasive approaches, is currently a challenge in diagnostic imaging.

### 1.3. Imaging of Peritoneal Carcinomatosis

Considering the poor prognosis associated with peritoneal dissemination, regardless of the disease’s stage, sensitive, specific, and non-invasive tools are needed to evaluate the presence of PC and to quantify its extent.

Ultrasound’s role in assessing peritoneal tumors is limited, but it proves valuable in detecting malignant ascites. Additionally, it represents an ideal modality for conducting image-guided biopsies when a histological diagnosis is necessary [14].

Multidetector computed tomography (CT) is frequently employed as the predominant imaging modality for evaluating the presence and extent of peritoneal disease and excluding extraperitoneal metastases, owing to its widespread availability and rapid acquisition capabilities. Nevertheless, numerous studies have demonstrated that this technique frequently underestimates the volume of peritoneal disease compared to surgical assessment [15,16].

One imaging technique, ^18^F-fluorodeoxyglucose positron emission tomography/computed tomography ([^18^F]FDG PET/CT), is a precise method for excluding nodal and extraperitoneal disease and identifying recurrences that might be overlooked on CT scans. However, it can yield inaccurate results, presenting false negatives in small peritoneal implants, mucinous tumors, or signet ring gastric cancers, as well as false positives in non-malignant inflammatory lesions [15,17].

Magnetic Resonance Imaging (MRI) is gaining prominence as a viable alternative imaging method for staging and surveillance. It offers higher soft tissue contrast and enables multiphasic contrast-enhanced and diffusion-weighted imaging. This capability is extremely valuable in detecting diseases in challenging areas such as the mesentery and the small bowel serosa. In addition to this, it is a radiation-free imaging modality that makes MRI particularly useful for therapy follow-up. Despite its well-known advantages, its main limitations include long examination times, motion artifacts, restricted applicability in patients with metal devices, and limited accessibility. Moreover, the interpretation of MRI findings strictly relies on the expertise of radiologists [14].

As previously mentioned, a noninvasive tool is crucial for both diagnosis and assessing cancer progression in ovarian cancer during follow-up; despite laparotomies still conventionally being considered the gold standard in the diagnosis of ovarian cancer recurrence, this invasive modality is more frequently associated with surgical complications compared to primary laparotomy [18,19].

The aim of this systematic review was to deeply assess the current state of art of imaging modalities for the evaluation of PC arising from malignant gynecological origins, with a focus on ovarian and endometrial cancers, highlighting the strengths and limitations of each imaging modality.

## 2. Materials and Methods

### Data Sources and Study Selection

A literature review was conducted utilizing the PubMed, EMBASE, and Scopus databases, encompassing data from the earliest available indexing dates to the last decade. A search algorithm based on the combined terms (ovarian cancer OR endometrial cancer) AND (peritoneal carcinomatosis) AND (computed tomography) AND (FDG) AND (MRI) was employed, complemented by manual searches. Only original papers published in English were considered, following the PRISMA 2020 guidelines [20]. Quality control was ensured by implementing the PRISMA checklist. Two authors independently critically assessed the overall quality of the selected studies. Diagnostic performance values (sensitivity, specificity, and accuracy) of different imaging techniques for detecting peritoneal carcinomatosis were extracted from each study. Only studies using surgery PCI as a gold standard were included.

## 3. Results

Following the comprehensive computerized search and cross-checking of reference lists, a total of 50 papers from PubMed, 61 from EMBASE, and 54 from Scopus were identified. After excluding duplicates, 74 papers were collected. Subsequently, two researchers reviewed titles and abstracts, excluding 39 papers that did not focus on specific data related to peritoneal carcinomatosis of gynecological origin. Additionally, nine case reports and four reviews were excluded. The same two researchers independently evaluated the full-text versions of the remaining 22 papers, excluding four due to insufficient data and seven without surgery PCI as a gold standard. Ultimately, 11 papers were included in the review of PCI surgery.

**Figure 3:**
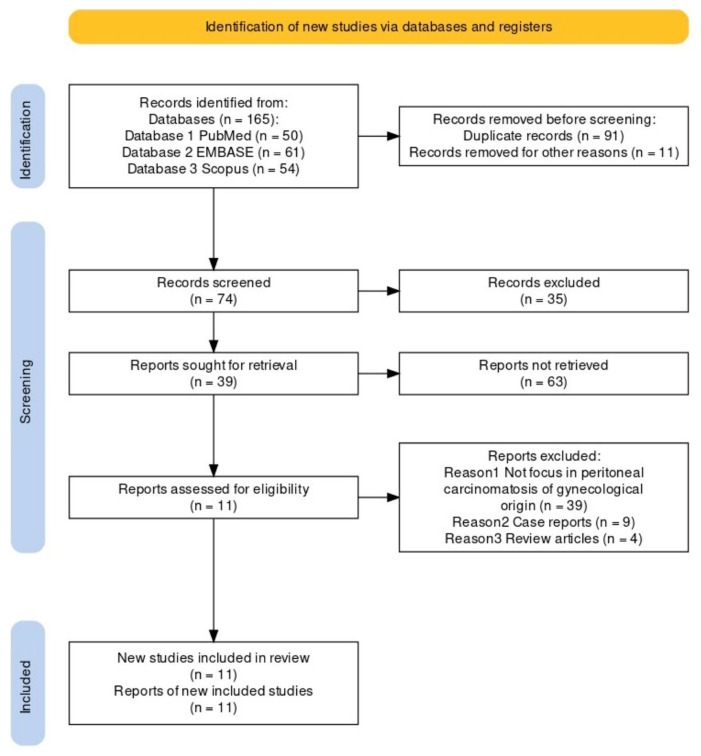
A flow chart depicting the search for eligible studies.

**Figure 4:**
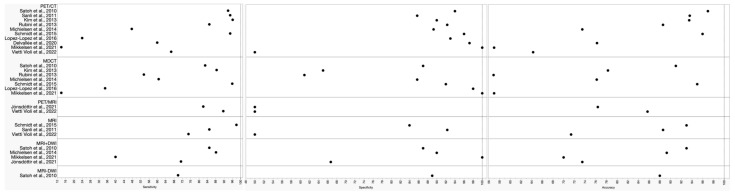
Overview of diagnostic performance of different imaging modalities reported in the eligible studies.

A flow chart depicting the search for eligible studies is shown in Figure 3. Information regarding study details including authors, publication year, study design, patient characteristics, technical aspects, sensibility, specificity, and accuracy was systematically collected (Table 1 and Figure 4).

Since very few papers reported in detail the values of true positives (TP), false positives (FP), true negatives (TN), and false negatives (FN) that are needed to calculate pooled sensitivity and specificity, a meta-analysis of the available literature could not be performed.

Ethics Committee approval was deemed unnecessary, as our study relied on published data.

## 4. Discussion

### 4.1. CT/CECT vs. [^18^F]FDG PET/CT

**Figure 5:**
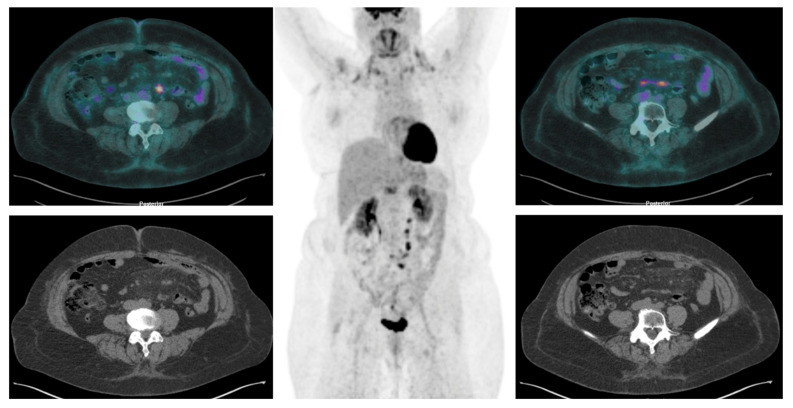
[^18^F]FDG PET/CT revealed a nodular hypermetabolic lesion in the mesenteric region and other foci of increased [^18^F]FDG accumulation in mesenterial soft tissue.

**Figure 6:**
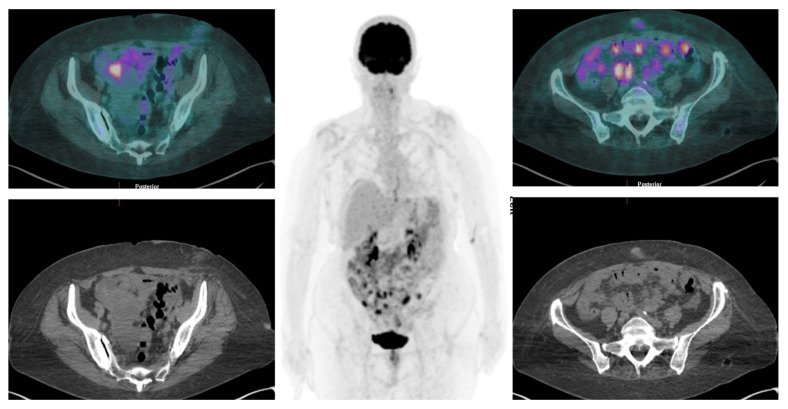
[^18^F]FDG PET/CT detects multiple nodular hypermetabolic lesions in the abdominopelvic region, suggestive of peritoneal carcinomatosis.

Over the past decade, numerous studies have highlighted the ability of [^18^F]FDG PET/CT over CT in detecting lymphadenopathies and peritoneal distant metastases associated with solid tumors (Figure 5 and Figure 6).

Several published studies have examined the diagnostic accuracy of PET/CT and contrast-enhanced CT (CECT) in assessing PC, with histological examinations serving as the gold standard. Nevertheless, these studies often involve small and heterogeneous patient groups and achieve controversial results [32,33].

Rubini et al. retrospectively compared [^18^F]FDG PET/CT and CECT in 79 patients with histologically confirmed ovarian cancer. PET/CT provided higher sensitivity, specificity, and accuracy in detecting PC, even in patients with ascites. However, in 2 out of 11 patients, [^18^F]FDG PET/CT failed to detect peritoneal lesions due to the mucinous histotype [24].

Kim et al. [23] reported higher values in terms of the sensitivity (96.2%), specificity (90.0%), and accuracy (93.5%) of [^18^F]FDG PET/CT compared to previous PET studies [24,32,34,35,36]. However, the authors did not directly compare the diagnostic performance of PET/CT and CECT simultaneously [23].

Numerous studies highlighted a correlation between the metabolic burden detected by PET/CT and the histological size, which strictly depends on tumor histotypes and behavior. For instance, standardized uptake values (SUVs) tend to be lower for ovarian mucinous cancers than for digestive tumors. However, in contrast, these mucinous cancers exhibit higher diffusion coefficients in MRI scans [37,38,39].

Lopez-Lopez et al. conducted a retrospective study comparing surgical PCI with the accuracy of preoperative PCI, assessed by PET/CT and CT, in 59 women with ovarian cancer and peritoneal spread of the disease and candidates for receiving CRS and hyperthermic intraperitoneal chemotherapy [27]. CT provided a sensitivity of 35%, a specificity of 98%, a positive predictive value (PPV) of 90%, and a negative predictive value (NPV) of 72%. In contrast, PET/CT exhibited a sensitivity of 24%, a specificity of 93%, a PPV of 66% and a NPV of 68%. It is well known that combining PET with CT enhances the anatomical localization of intra- and extra-pelvic structures. This integrated approach also provides more reliable information concerning the nature of pathological findings. Nevertheless, in their study, Lopez-Lopez et al. concluded that the primary clinical advantage of [^18^F]FDG PET/CT is in assessing the extraperitoneal extent of diseases rather than peritoneal involvement, where CT shows better performance [27]. These results are consistent with the conclusions provided by Funicelli et al. and Hynnimen et al.; both observed no significant advantages in integrating [^18^F]FDG PET/CT into the preoperative assessment of patients when compared to the outcomes derived from CT imaging [35,40].

In 2020, Delvallée et al. conducted the first multicenter prospective study using a PET/CECT protocol. The study involved 90 women with epithelial ovarian cancer diagnosed by [^18^F]FDG PET/CT scan before undergoing surgery or any other treatments. Their findings showed that the correlation between [^18^F]FDG PET/CT PCI and surgical PCI was more favorable for endometrioid and mucinous types, aligning with studies on digestive neoplasia [28]. However, as mentioned in the literature, the histological type impacts SUVs, with higher values observed in cases of endometrioid histotypes [37,38,39]. Moreover, the effectiveness of [^18^F]FDG PET/CT may differ according to the tumor size of peritoneal lesions and their location [28].

PET/CT during NACT enables the assessment of treatment effectiveness and guides clinicians to evaluate tumor residue post-treatment. However, the authors found lower values for the sensitivity and specificity of [^18^F]FDG PET/CT compared to those reported in other studies and meta-analyses [41,42,43,44].

### 4.2. MRI ± DWI vs. FDG PET/CT

MRI is widely recognized as a crucial technique for evaluating malignant tumors. Advances in MRI technology have enabled high-b-value diffusion-weighted imaging (DWI) performance with an enhanced signal-to-noise ratio. This improvement allows for the accurate detection of malignant tissue. Several studies have explored the role of MRI and DWI in examining the peritoneal dissemination of solid tumors.

In 2015, Schmidt et al. conducted a pioneering study that prospectively compared the diagnostic capabilities of multidetector contrast-enhanced CT (MDCT), MRI, and [^18^F]FDG PET/CT in the examination of 15 women with PC during primary ovarian cancer staging. The study revealed no significant differences between MDCT, MRI, and PET/CT, showing a sensitivity of 96%, 98%, and 95% and a specificity of 92%, 84%, and 96%, respectively. They, therefore, concluded that the three modalities are comparable but, given the fastest execution and widest availability of MDCT, compared to MRI and PET/CT, MDCT is the modality of choice when a stand-alone modality is needed [26].

Sanli et al. conducted a study comparing PET/CT and conventional MRI without DWI sequences for detecting recurrent ovarian cancer. Their findings showed that both PET/CT and MRI had similar sensitivity in detecting recurrent ovarian cancer, with rates of 95% and 85%, respectively. However, PET/CT exhibited higher overall accuracy, standing at 93.6% compared to 88.6% of MRI. Moreover, since the detection of peritoneal implants typically relies on their size and the presence of ascites, the authors concluded that PET/CT perform better than MRI in patients with small-to-medium-size (<2 cm) peritoneal implants [22].

Satoh et al. conducted a retrospective study in patients exhibiting clinical suspicion of abdominal peritoneal tumor dissemination. The study included 170 patients who underwent PET/CT and 130 who underwent CECT and MRI [21]. The assessment of and ADC from MRI with DWI was not conducted in this study. No significant differences in the sensitivities and specificities of [^18^F]FDG PET/CT, CECT, and MRI + DWI were observed, but [^18^F]FDG PET/CT showed a notably higher PPV compared to MRI + DWI. They concluded that MRI might effectively detect peritoneal involvement by analyzing T1- and T2-weighted images without DWI. However, high-b value MR images were acquired by including DWI. The most notable limitation of this study was the exclusion of ADC values in the image analysis. Indeed, as the authors also suggest, incorporating ADC measurements could potentially enhance the PPV of MRI + DWI, as demonstrated in previous studies [45,46,47].

Michielsen et al. assessed the effectiveness of whole-body MRI with DWI (WB-DWI/MRI) in staging and in determining operability in patients with suspected ovarian cancer, comparing to CT and [^18^F]FDG PET/CT. A non-PCI region-based analysis found that DWI-MRI was 89.3% accurate in peritoneal staging. In comparison, CT scored 76.2% and [^18^F]FDG PET/CT scored 73.5% [25].

Mikkelsen et al. conducted a prospective study comparing the effectiveness of DW-MRI, CT, and [^18^F]FDG PET/CT in assessing tumor presence and potential involvement in critical anatomical areas before surgery in 50 patients with advanced-stage epithelial ovarian cancer. The study revealed that PCI assessment via imaging techniques was frequently undervalued across all three modalities compared to surgical findings. Despite significant overall differences among the modalities in determining PCI, DW-MRI emerged as the most accurate, especially in cases with a substantial tumor burden where surgical PCI exceeded 20 [29].

### 4.3. [^18^F]FDG PET/MRI

The [^18^F]FDG PET technique combined with MRI has emerged as an innovative hybrid imaging technique. Its application has been explored in the study of primary head and neck cancer, colorectal cancer, and primary gynecologic malignancies [48,49]. The integration of PET and MRI presents potential advantages compared to PET/CT, owing to the superior morphological capabilities of MRI and its additional contribution of functional information through techniques such as DWI. This combination offers a comprehensive approach integrating anatomical and functional data, presenting a promising avenue for enhanced diagnostic capabilities [49,50].

Catalano et al. evaluated the possible role of [^18^F]FDG PET/MRI and stand-alone MRI for the staging of PC originating from colorectal cancer in 62 untreated patients. In their series, PET/MRI was superior to MRI in evaluating lesion size, in assessing N status, and in determining external sphincter infiltration [48]. Queiroz et al. compared PET/MRI and PET/CT in both the staging and restaging of gynecological tumors and observed a superiority of PET/MRI in assessing primary tumors and no significant differences in the assessment of peritoneal involvement in 26 patients with primary ovarian cancer [49].

Two recent papers have investigated the use of [^18^F]FDG PET/MRI in patients with PC originating from gynecologic malignancies [30,31]. In 2021, Jonsdottir and colleagues conducted a prospective study to validate the use of [^18^F]FDG PET/MRI compared to DW-MRI alone. They found that both DW-MR- and [^18^F]FDG PET/MR-derived PCI were highly and positively correlated with the surgical PCI (DW-MR: β = 0.86 ± 0.14 *p* < 0.01, [^18^F]FDG PET/MR: β = 0.94 ± 0.01 *p* < 0.01) [30]. The author analyzed various abdominal regions, and the findings revealed that PET/MR demonstrated greater sensitivity for detecting carcinomatosis than DW-MRI in small bowel regions. Both [^18^F]FDG PET/MRI and DW-MRI-determined total PCI correlated well with the gold standard surgical PCI, showing [^18^F]FDG PET/MRI as having a notably higher correlation with the total operative PCI compared to DW-MRI alone. In assessing patients at initial diagnosis, [^18^F]FDG PET/MRI demonstrated greater accuracy than DW-MRI. However, no significant difference was observed in patients undergoing prior chemotherapy. The study also highlighted the superior performance of [^18^F]FDG PET/MRI in individuals deemed inoperable, with a substantial tumor burden [30]. In 2022, Vietti Violi et al. confirmed these findings. In their study, [^18^F]FDG PET/MRI demonstrated adequate diagnostic accuracy in identifying patients with a significant tumor burden, achieving a remarkable 100% sensitivity and specificity when using a cut-off PCI of 21. Compared to standalone MR or [^18^F]FDG PET, the combination of [^18^F]FDG PET/MRI yielded enhanced sensitivity (91.7%) compared with MRI (75%) and [^18^F]FDG PET (66.7%) [31].

Earlier researchers [51,52] have emphasized the advantages of hybrid [^18^F]FDG PET/MRI systems in enabling the simultaneous acquisition of both PET and MRI signals to minimize moving phenomena. This becomes significant in the abdomen and mesentery, where organ positions can change due to peristaltic motion. This specific capability presents an advantage over [^18^F]FDG PET/CT, which follows a sequential acquisition method. This proves especially valuable in examining carcinomatosis, a condition often found in the mesentery. [^18^F]FDG PET/MRI offers an added benefit compared to [^18^F]FDG PET/CT due to the lack of radiation exposure.

## 5. Strengths and Limitations

Data in the literature on the use of hybrid imaging has increased over the last few decades, but in our systematic review, there emerged a wide variability in the reported diagnostic performance of different imaging techniques. Amongst these, technical issues such as the different equipment used by each individual study (e.g., type of tomograph), different acquisition protocols (e.g., the application of specific sequences in MRI or PET/MRI studies, CT or CTE co-registration), and interpretation criteria are the main sources.

In addition to this, heterogeneity in patient populations ([^18^F]FDG avidity related to histotypes, disease stage, prior therapies) may also explain this variability. Nevertheless, the influence of chemotherapy prior to imaging cannot be deeply assessed yet, given the paucity of available data.

Although it has not been possible to perform a meta-analysis and a cost–benefit analysis on the available literature for the above-mentioned reasons, the results of this systemic review show that the combination of morphological imaging techniques, such as CT and MRI with [^18^F]FDG PET, may improve the evaluation of PC in gynecological tumors.

## 6. Implications for Clinical Practice

Imaging plays a crucial role in both the staging and restaging of several cancers, and progressive improvements in technology are contributing to changing diagnostic approaches in oncology, as well as in other fields. In particular, hybrid imaging offers the possibility of studying PC from both an anatomic and metabolic point of view, thus allowing a non-invasive and precise functional characterization of neoplastic foci. Moreover, [^18^F]FDG PET/CT, CT and (PET/)MRI allow for the obtaining of accurate information on disease location and extent to a level very close to that obtained at surgical laparoscopy, thus playing a crucial role in patient management.

## 7. Conclusions

A wide variability of reported accuracies for [^18^F]FDG PET/CT, MRI, and PET/MRI in the assessment of PC still emerges from the available literature, but overall, this systematic review highlights the crucial role of hybrid imaging in assessing PC arising from gynecological tumors. In many published comparative studies, a clear superiority of a particular imaging modality has not emerged so far, thus suggesting that they can be all used complementarily to surgical laparoscopy.

Larger prospective and retrospective studies comparing [^18^F]FDG PET/CT, [^18^F]FDG PET/MRI, CT, and MRI in more homogeneous patient populations are needed in order to perform a meta-analysis and to definitively identify the most accurate imaging modality for diagnosing PC in gynecological cancers. This could be very relevant for further improving the management of these patients.

## Figures and Tables

**Table 1:** Overview of selected studies.

Study	Year	Study Design	Patient Number	Image Modality	Age	Primary Cancer	Sensitivity	Specificity	Accuracy
Satoh et al. [21]	2010	Retrospective	107 130 130 130	PET/CT	29–89	Ovarian and colon cancer	94.0	94.0	97.0
				MDCT			83.0	87.0	91.0
				MRI + DWI			85.0	87.0	93.0
				MRI-DWI			70.0	89.0	88.0
Sanli et al. [22]	2011	Retrospective	47 47	PET/CT	38–78	Ovarian cancer	95.0	85.7	93.6
				MRI			85.0	92.31	88.61
Kim et al. [23]	2013	Retrospective	46 46	PET/CT	27–78	Ovarian cancer	96.2	90.0	93.5
				MDCT			88.5	65.0	78.3
Rubini et al. [24]	2012	Retrospective	79 51	PET/CT CECT	30–83	Ovarian cancer	85.0	92.3	88.6
							53.6	60.9	56.9
Michielsen et al. [25]	2014	Prospective	32 32 32	PET/CT	20–83	Ovarian cancer	47.8	89.3	73.5
				MDCT			60.7	85.7	76.2
				MRI + DWI			88.2	90.0	89.3
Schmidt et al. [26]	2015	Retrospective	15 15 15	PET/CT	31–89	Ovarian cancer	95.0	96.0	96.0
				MDCT			96.0	92.0	95.0
				MRI			98.0	84.0	93.0
Lopez-Lopez et al. [27]	2016	Retrospective	59 59	PET/CT	27–78	Ovarian cancer	24.0	93.0	-
				MDCT			35.0	98.0	-
Delvallée et al. [28]	2020	Retrospective	90	PET/CT	63	Epithelial ovarian cancer	60.0	97.2	76.3
Mikkelsen et al. [29]	2021	Prospective	50 50 50	PET/CT	32–78	Epithelial ovarian cancer	14.0	100	57.0
				MDCT			14.0	100	57.0
				MRI + DWI			40.0	100	70.0
Jónsdóttir et al. [30]	2021	Prospective	34 34	MRI + DWI PET/MRI	37–78	Gynecological cancer	71.4	66.7	73.5
							82.1	50.0	76.4
Vietti Violi et al. [31]	2022	Prospective	14 14 14	PET/MRI	45–69	Peritoneal carcinomatosis	91.7	50.0	85.7
				MRI			75.0	50.0	71.4
				PET			66.7	50.0	64.3

## Data Availability

Data are available upon request.
